# Reply: Lecanemab: turning point, or status quo? An ethics perspective

**DOI:** 10.1093/brain/awad102

**Published:** 2023-03-30

**Authors:** John Hardy, Catherine Mummery

**Affiliations:** Reta Lilla Weston Research Laboratories, UCL Institute of Neurology, London WC1N 3BG, UK; Department of Neurodegenerative Disease, UCL Institute of Neurology, London WC1N 3BG, UK; Dementia Research Institute, UCL Institute of Neurology, London WC1N 3BG, UK; Department of Neurodegenerative Disease, UCL Institute of Neurology, London WC1N 3BG, UK

We are pleased to respond to Dr Daly’s critique of our commentary^1^ on anti-amyloid trials.^2^ We were surprised that he deemed such trials ‘unethical’: this is both legally suspect, as all of them are, of course, supported by informed consent and approved and monitored by rigorous ethics committees, and also marginally insulting to the trialists and their patients who are working together to try and reduce the burden of this terrible disease.

He makes the point, to which we also alluded, that there have been many previous and failed ‘anti-amyloid’ trials. There are many reasons for this: some purported ‘anti-amyloid trials’ did not hit their target^3^ and thus were not genuinely anti-amyloid trials, and many earlier trials did not remove amyloid, but rather prevented further amyloid build up which we now realise was not enough.^4^ The value, however, of these earlier trials was that it was through their analysis we learned the hard fact that to be successful, drugs had to remove amyloid.

As to the changing nature of the amyloid hypothesis^1,5^: of course, we acknowledge this. There would be no point in doing experiments if we did not allow the data from these experiments (both basic and clinical) to change our mind. As J. K. Galbraith is reported to have said ‘When the facts change, I change my mind—what do you do?’.

We hope that the positive lecanemab data is the first swallow of spring for Alzheimer therapeutics and that other, and perhaps more effective and easier to use drugs and interventions are now developed that are aimed both against amyloid and other biologically validated targets. Clearly, every researcher working on Alzheimer’s disease and as well as in patient support groups, wants to reduce the burden of this disease and double blind, placebo-controlled trials, backed by informed consent, are the most effective way we have to achieve this aim.

## Data Availability

Data sharing is not applicable to this article as no new data were created or analysed in this article.
